# Air Pollution: It’s Time for a New Deal to Protect Health. Statements of the Spanish Ministry of Health at the 2019 Global Climate and Health Forum in Madrid

**DOI:** 10.3390/ijerph17030825

**Published:** 2020-01-28

**Authors:** María Luisa Carcedo Roces

**Affiliations:** Minister of Healthcare, Consumer Affairs and Social Welfare Government of Spain, 28014 Madrid, Spain; secmin@mscbs.es

With each inspiration, the oxygen from the inhaled air gets into the lungs, and it is adsorbed. The oxygen-rich blood is then carried to the heart and distributed throughout the body. At the same time, in the lungs, the blood removes carbon dioxide, which is exhaled into the atmosphere through each exhalation. Humans, as with all other mammals, can live thanks to this essential process. We can breathe air from 8 to 16 times per minute for adults, and up to 44 times per minute for babies.

However, instead of “clean air”, we are now breathing polluted air, which is already damaging human health, and at the same time has a long-term impact. Scientific evidence has already shown that air pollution is responsible for a significant number of deaths and hospitalizations. It also causes numerous diseases and worsens symptoms of pre-existing diseases. The evidence has also shown that the impact of climate change on health is not neutral. There are vulnerable groups for which pollution has a greater impact, such as children, elderly people, pregnant women, sick people and those with low socioeconomic status. We know that the impact on children´s health occurs even at lower concentrations of air pollution than in the case of adults. Boys and girls who are exposed to high levels of air pollution are therefore at a high risk of developing chronic diseases such as asthma or cardiovascular illnesses. The effects on intrauterine growth should also not be overlooked: air pollution can lead pregnant women to premature births, and result in newborns who are smaller or underweight. In addition, air pollution can impair the neurological development and cognitive capacity of boys and girls.

With this cause-and-effect relation in mind, some information is presented below:

According to the World Health Organization (WHO), air pollution causes seven million premature deaths each year, of which 600,000 are children.

Worldwide, more than 90% of the child population lives in environments with levels of air pollution which are above WHO guidelines.

Cities are hot-spots of air pollution, and it is estimated that by 2050, 70% of the world’s population will live in cities.

Living near high-traffic roads is responsible for 15% of childhood asthma and a similar percentage of chronic diseases in people over 65 years.

The consequences of climate change are already a public health emergency. Climate change is an environmental disaster and an emergency for human health. I have only mentioned the health impact of one factor—air pollution—but there are many other climatic threats that also affect health: the increase in average temperatures and days of extreme heat; extreme weather events such as fires, floods, and droughts; the risks to seafood security; malnutrition linked to crop alterations; or the increase in mosquito-borne diseases, some of which were considered to have been eradicated.

This reality is no longer an apocalyptic dystopia; it is very palpable, “breathable”, and, unfortunately, has turned into an everyday reality. We cannot continue without dimensioning/appraising the terrible threat to human health posed by climate change. It is time to act now, and it is time to talk about the Health New Deal.

According to the conclusions of the 2019 Lancet Countdown Report, we are being slow to respond to the huge impact that climate change has on health. Inactivity has a cost that we cannot afford. Our response must be multilateral and multisectoral. It must be firm and urgent, and it must involve international organizations, governments, civil society organizations, research institutions, private sector, and the citizenship.

At the Ministry of Healthcare, Consumer Affairs and Social Welfare of the Government of Spain, we are committed to this joint response. In the coming months, we will present a National Health and Environment Plan, elaborated with the participation of other Ministries, regional governments and the best experts, with an approach that advocates including “health in all policies”. This plan contains our commitments to the European Union and to the international organizations, particularly the WHO.

The Spanish National Health and Environment Plan will describe the main environmental factors that affect human health and will establish the objectives and lines of intervention related to a) the impact on health of air quality, habitat and water; and b) the management of products and chemical risks, endocrine disruptors, biomonitoring, environmental radioactivity, electromagnetic fields and non-ionizing radiation, UV radiation or noise, among other aspects.

In recent years, civil society has been able to convey the negative effects of climate change on the planet and now must also be an ally to deliver the message of its impact on health. The scientific evidence is overwhelming and, as pointed out by researchers from the Carlos III Health Institute during the COP25 in Madrid, scientists need the help of the media and civil society to claim the prominence that health should have when we talk and act on climate change. 

Companies, including those operating in the health sector, are also slow to link health and climate change. According to The Lancet Countdown, out of the 12,000 companies (from more than 160 countries) that have signed the United Nations Global Compact agreement to achieve Sustainable Development Goals by 2030, only 12% have linked health and climate change in their communications progress reports. 

The Health New Deal cannot wait any longer. It is time to act now because few things are as essential as the air we breathe. Evidence is telling us that we must act urgently. We cannot allow ourselves any delay or setback in progress, because our sons and our daughters will not forgive us. Only by placing health at the center of all policies and actions will we achieve effective commitments that will allow us to save lives, reduce polluting emissions, breathe clean air, and ultimately promote fair and sustainable development.

